# “NO LOAD” Resistance Training Promotes High Levels of Knee Extensor Muscles Activation—A Pilot Study

**DOI:** 10.3390/diagnostics10080526

**Published:** 2020-07-29

**Authors:** Rafael Ribeiro Alves, Carlos Alexandre Vieira, Martim Bottaro, Murilo Augusto Soares de Araújo, Daniel Costa Souza, Bruno Cavalcante Gomes, Paulo Gentil

**Affiliations:** 1 Faculdade de Educação Física e Dança, Universidade Federal de Goiás, Goiania 74690-900, Brazil; alves.rafael.ribeiro@gmail.com (R.R.A.); vieiraca11@gmail.com (C.A.V.); contato.muriloaugusto@gmail.com (M.A.S.d.A.); daniel_souza86@hotmail.com (D.C.S.); bruno.c.gomes@hotmail.com (B.C.G.); 2 Faculdade de Educação Física, Universidade de Brasília, Brasília 70910-900, Brazil; martim@unb.br; 3 Liga de Hipertensão Arterial, Universidade Federal de Goiás, Goiania 74605-020, Brazil

**Keywords:** resistance exercise, muscle activation, strength training, rehabilitation, motor unit activation

## Abstract

The present article aims to compare electromyographic (EMG) activity of the knee extensors during traditional resistance training (TRT) and no load resistance training with or without visual feedback (NL-VF and NL-NF). Sixteen healthy men (age: 25.2 ± 3.6) volunteered to participate in the study. Participants visited the laboratory on three occasions involving: (1) a 10 repetition maximum test (10 RM test), (2) familiarization and (3) performance of knee extensions using TRT, NL-VF and NL-NF in a random order, with 10 min of rest between them. TRT involved the performance of a set to momentary muscle failure using the 10 RM load. NL-NF involved the performance of 10 repetitions with no external load, but with the intention to maximally contract the muscles during the whole set. NL-VF involved the same procedure as NL-NF, but a monitor was positioned in front of the participants to provide visual feedback on the EMG activity. Peak and mean EMG activity were evaluated on the vastus medialis (VM), vastus lateralis (VL) and rectus femoris (RF). Results: there were no significant differences in VM and VL peak EMG activity among different situations. There was a significant difference for peak EMG activity for RF, where TRT resulted in higher values than NL-VF and NL-NF (*p* < 0.05). Higher values of mean EMG activity were found for VM, VL and RF during TRT in comparison with both NL-VF and NL-NF. Conclusions: resistance training with no external load produced high levels of peak muscle activation, independent of visual feedback, but mean activation was higher during TRT. These results suggest that training with no external load might be used as a strategy for stimulating the knee extensors when there is limited access to specialized equipment. Although the clinical applications of no load resistance training are promising, it is important to perform long-term studies to test if these acute results will reflect in muscle morphological and functional changes.

## 1. Introduction

Resistance training is one of the most popular forms of physical exercise and commonly aims to increase muscle strength and mass [1,2,3]. It has been used to promote benefits in a wide range of populations, including healthy young people and chronically ill patients [4,5,6]. The external load used during resistance training is considered to be one the main factors to achieve the desired results [7]. The American College of Sports Medicine [3] recommends that an external load of at least 60% of one repetition maximum (1 RM) should be used for novice and intermediate trained people with the purpose to increase muscle strength and size.

However, the results of some studies question the importance of using high external loads. When effort is matched, studies involving different margins of loads and repetition lead to similar gains in muscle strength and size [8,9,10] and there is evidence that external loads as low as 30% of 1 RM might promote significant results in these outcomes [9,11]. This effort based approach opens the possibility of performing resistance training using non-traditional approaches and have the same results as during traditional resistance training (TRT) such as elastic bands [12,13,14], body weight exercises [15,16] and even some training models traditionally associated with aerobic activities such as cycling [9,17,18]. This evidence raised the suggestion that effort, rather than external load, might be a key determinant of training adaptations [8,10,19,20].

In agreement with this suggestion, Counts et al. [20] found similar gains in muscle size when comparing two distinct training protocols in young men. The study involved a contralateral design in which one arm performed traditional resistance training (TRT) with 70% of 1 RM, while the other trained without external load but tried to maximally contract the muscles during the full range of motion. The results showed that the elbow flexors’ muscle thickness increased similarly in both situations. Both protocols resulted in significant increases in one repetition maximum (1 RM); however, the increases were higher for TRT. Later, Barbalho et al. [21] compared the effects of training with no external load and training with elastic bands for the upper body muscles in hospitalized patients. The study involved twenty hospitalized patients and the results showed that biceps brachii, triceps brachii and pectoralis muscle thickness similarly increased in both groups, as well as functional performance measured by the 30 s elbow flexion test. These results extended the perspective brought by previous studies, demonstrating that neuromuscular adaptations such as increased muscle size and strength can occur even in the absence of external load, provided that muscle fibers are sufficiently stressed.

The practical applications for training with no external load are promising since specialized equipment might not be available in many situations, such as during social isolation, travelling and hospitalization. However, one important limitation of the previous studies is that they involved only upper body muscles. Whilst upper body muscles might be important for daily activities (personal care, feeding, etc.), it would be important to extend these findings to lower body muscles due to its importance in functionality and locomotion [22,23]. Moreover, there is evidence that lower body muscles respond differently than upper body muscles and there seems to be a greater difficulty to train theses muscles with higher efforts [24], which might explain why they usually benefit from higher training volumes [25,26].

Therefore, studies evaluating the effects of resistance training with no external load on lower body muscles are needed to provide further insights into this strategy. One important step before proceeding to long-term studies is to analyze the acute effects of the interventions in a pilot study, so researchers can have more consistent information to perform future studies and evaluate their rationale and methodological aspects. The positive results obtained from resistance training with no external load might be associated with the high level of effort employed, reflected by the high levels of muscle activation [21,27]. Therefore, the analysis of this acute response might be important to get insights about its effects and provide rationale for long-term studies. The aim of this pilot study was to compare the effects of TRT and no load resistance training on the knee extensor muscle activity of young men.

## 2. Materials and Methods

### 2.1. Study Design

**Figure 1:**
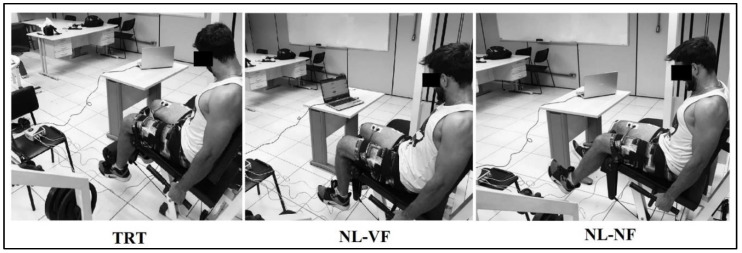
Experimental conditions. TRT, traditional resistance training, NL-VF, no load with visual feedback; NL-NF, no load with no feedback.

The experiment involved three visits. The first session involved anthropometric measures and 10 repetitions maximum (10 RM) tests in the leg extension exercise. After 72–96 h, in the second session, the volunteers participated in a familiarization session, where the procedures were explained and trials were performed at a lower intensity, to ensure compliance with the proposed movement velocity. In the third session, after 72–96 h, muscle activity was measured while the participants performed traditional resistance training (TRT) and no external load resistance training with visual feedback (NL-VF) or without visual feedback (NL-NF). The protocols were performed in a randomized order and separated by 10 min intervals. Electrodes were fixed on the vastus medialis (VM), lateralis (VL) and rectus femoris (RF) muscles to evaluate the peak and mean electromyographic (EMG) activity as illustrated in Figure 1.

### 2.2. Subjects

A priori sample analysis revealed that to achieve a 0.5 effect size (ES) with a power of 0.8 and significance of 0.05 a total of nine participants would be necessary. Therefore, sixteen healthy men were recruited to account for eventual attrition. The characteristics of the participants are described in Table 1. To participate, volunteers had to have previous experience in the leg extension exercise (≥2 months of uninterrupted practice). The participants did not report any physical limitations, health problems or musculoskeletal injuries that could have affected or be aggravated by the tests. None of the participants were taking medications or substances expected to affect physical performance. After being informed of the experimental procedures and all possible risks and discomforts related to the study protocol, subjects signed an informed consent form approved by the local Institutional Ethics Committee (approval number: 56907716.5.0000.5083 in October 2017). The present study was performed in accordance with the Declaration of Helsinki.

### 2.3. Ten Repetitions Maximum (10 RM) Test

Participants performed 10 RM tests on the leg extension exercise (Rotech Fitness, Goiania, Brazil). Before the tests, the participants warmed up with 10 reps at a comfortable self-selected load and then rested for 5 min. The initial load was defined based on the participants’ training history. If the volunteer could not perform 10 repetitions or performed more than 10 repetitions, the load was adjusted by 1–10 kg and another attempt was performed after 5 min of rest. No more than three attempts were necessary on any occasion. The concentric and eccentric phase of each repetition was controlled with the support of a metronome and lasted 2 s each. Verbal encouragement was provided during all tests and the test was interrupted when the participant was not able to follow the determined cadence [27].

### 2.4. Experimental Procedures

The training protocol consisted of one set of knee extensions in each situation, performed in a randomized order and with an interval of 10 min between them.

- TRT involved the performance of a set to momentary muscle failure, as previously defined [19], using the 10 RM load and with the same procedures described in the 10 RM test. The repetitions were performed with a predetermined cadence of 2 s for both concentric and eccentric phases and no pause between phases. The set was interrupted when the participant was unable to adhere with the stablished cadence [27] or could not move the load.
- NL-NF involved the performance of 10 repetitions with no external load. The exercise was performed with the intention to maximally contract the muscles during the whole set at a cadence of 2 s for both the concentric and eccentric phases, with no rest between phases.
- NL-VF involved the same procedure as NL-NF, but a monitor was positioned in front of the participants to provide visual feedback on the EMG activity.

Verbal stimulus was provided in all sets by the same researcher to maximize exercise performance and a metronome was used to help control the velocity.

### 2.5. Electromyographic Activity

**Figure 2:**
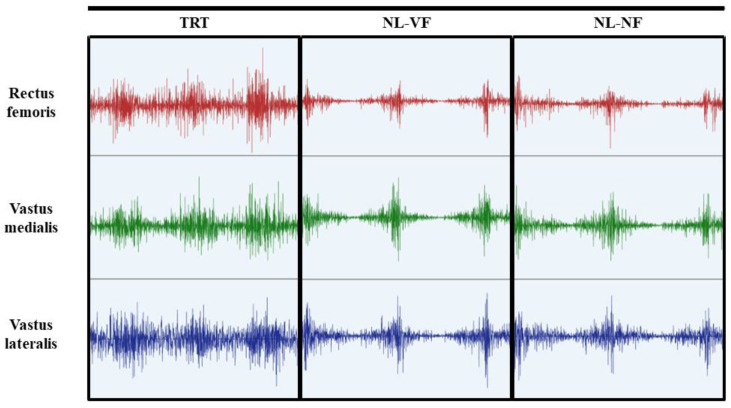
Illustration of electromyographic signal record. TRT, traditional resistance training, NL-VF, no load with visual feedback; NL-NF, no load with no feedback.

EMG activity was recorded for the VM, VL and RF. After trichotomy pairs of electrodes were positioned in a bipolar configuration (Distance from 20 mm between electrodes) along the direction of the muscular fibers, according to the recommendations of the SENIAM (www.seniam.org). The reference electrode was positioned on the patella. The identification of anatomical points and electrode placement were performed by the same researcher. EMG activity was measured using an EMG system with four channels (Miotool 400, Resolution of 14 bits, Miotec-Biomedical Equipment, Porto Alegre, Brazil) and with a sampling frequency of 2000 Hz per channel. After measurement, the EMG signals were filtered using the Butterworth filter with 20 Hz and cutoff frequencies of 500 Hz for the lower and upper band, respectively. Peak EMG activity was recorded as the peak value (µVs) obtained during each protocol. The percentage value of the mean activity EMG was normalized using as reference the maximum value (peak) obtained during the tests, considering the three protocols (Figure 2).

### 2.6. Statistical Analysis

Data normality was tested using the Shapiro–Wilk test. Depending on data normality, one-way repeated measures analysis of variance (ANOVA) or Friedman’s test were used to compare peak and mean EMG values among different exercise conditions (i.e., TRT, NL-NF and NL-VF). Post-hoc comparison with Bonferroni’s adjustment was used when necessary. The degrees of freedom were corrected by Greenhouse–Geisser estimates when sphericity hypothesis was violated. All analyses were performed using the Statistical Package for the Social Sciences (SPSS, Chicago, IL, USA) version 20.0. The results are presented as mean ± standard deviation and the accepted level of significance was *p* ≤ 0.05.

## 3. Results

**Figure 3:**
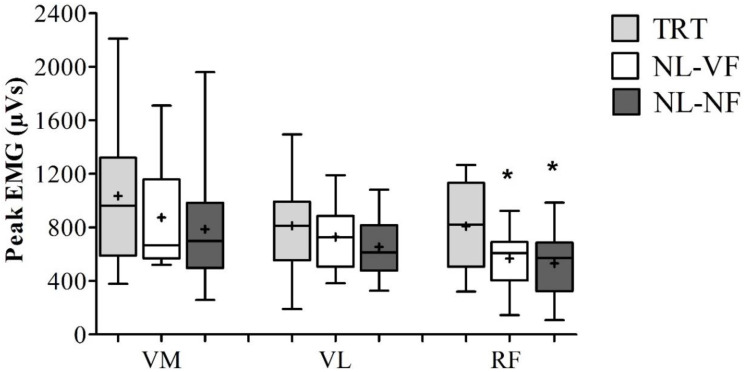
Comparison of peak EMG activity between trainings. TRT = traditional resistance training; NL-VF = no-load training with visual feedback; NL-NF = no load training without visual feedback; VM = vastus medialis; VL = vastus lateralis; RF = rectus femoris. Values are presented as median (lines) with interquartile range (boxes) ± range (minimum and maximum) and + indicates mean * *p* < 0.05 vs. TRT.

Friedman’s test showed no differences in VM peak EMG activity between TRT, NL-VF and NL-NF (961.3 ± 130.2, 697.7 ± 108.3 and 666.6 ± 103.6 mVs, respectively) (χ^2^2) = 4.625; *p* = 0.10). One-way ANOVA demonstrated similar results in VL peak EMG activity, with no significant differences between TRT, NL-VF and NL-NF (812.3 ± 349.3, 654.2 ± 227.1 and 729.1 ± 242.4, respectively) (F(1.459, 21.892) = 3.145; *p* = 0.08). However, TRT resulted in higher RF peak activity (809.7 ± 322.2) compared with NL-VF (531.1 ± 251.8) and NL-NF (567.5 ± 222.0). There was no difference between NL-VF and NL-NF peak EMG activity for any muscle (Figure 3).

**Figure 4:**
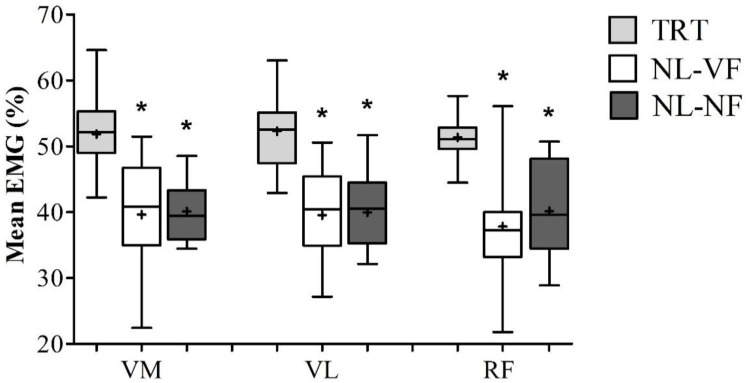
Comparison of mean EMG activity between different training protocols. TRT = load resistance training; NL-VF = visual feedback resistance training; NL-NF = no load resistance training; VM = vastus medialis; VL = vastus lateralis; RF = rectus femoris. Values are presented as median (lines) with interquartile range (boxes) ± range (minimum and maximum) and + indicates mean * *p* < 0.05 vs. TRT.

When comparing the mean EMG values, one-way ANOVA demonstrated higher levels during TRT for VM when compared with NL-VF and NL-NF (51.8 ± 5.4, 39.6 ± 7.9 and 40.1 ± 4.8%, respectively) (F(1.439, 21.579) = 21.060; *p* = 0.00), as well as for VL (52.3 ± 5.9, 39.5 ± 6.8 and40.1 ± 7.7%, respectively) (F(2.30) = 20.818; *p* = 0.00) and RF (51.3 ± 3.4, 37.8 ± 8.6 and 40.2 ± 7.7%, respectively) (F(2.30) = 28.482; *p* = 0.00). There was no difference between NL-VF and NL-NF for any muscle (Figure 4).

## 4. Discussion

The aim of the present study was to compare EMG activity of knee extensor muscles during TRT, NL-VF and NL-NF. The results demonstrated similar responses for VM and VL muscles’ peak EMG activity among the protocols. However, TRT resulted in higher RF peak EMG activity than both no load situations. In addition, TRT resulted in higher mean EMG values for all muscles analyzed.

Our findings are in agreement with previous studies that reported high levels of muscle activation during no load resistance training [21,27] for the arm muscles in many different situations. Counts et al. [20] reported high levels of biceps brachii muscle activation during NL-VF in untrained men and women. Later, Gentil et al. [26] reported that no load resistance training promoted high levels of muscle activation in young men independent of training status (trained vs. untrained), movement velocity, visual feedback and muscles analyzed (biceps and triceps brachii). This study also analyzed both peak and mean EMG activity; however, it did not compare muscle activation between TRT and no load resistance training. Considering that both studies [21,27] involved upper limb exercises, the present results extend the observation to lower body muscles and brings information for the possible use of no load resistance training to other body parts.

It is important to note, however, that peak RF activation during TRT was higher than both no load conditions. This is possibly because RF is a biarticular muscle with a complex musculotendinous architecture [28], which can result in an active insufficiency during knee extension. Therefore, the external load provided by TRT, especially when the knees are flexed and during eccentric actions, might have increased muscle activation, as suggested by Maeo et al. [29].

In the present study, mean EMG values were higher during TRT than both NL-VF and NL-NF. In a previous study by Counts et al. [20], the biceps EMG amplitude was similar for TRT and NL-VF elbow flexions for the first three repetitions; however, biceps muscle activation of the last three repetitions had greater amplitude for TRT than NL-VF. This suggests that it might be easier to exert continuous maximum contraction during elbow flexions than knee extensions, at least in the beginning of the exercise. It is important to highlight that in the protocol used by Counts et al. [20] the amount of repetitions performed during NL-VF was approximately twofold higher than TRT (20 vs. 8–12 repetitions). Therefore, the decrease in muscle activation in the last repetitions might be a consequence of the difficulty in sustaining high efforts for a prolonged time. In this regard, it seems likely that the manipulation of other RT variables such as the number of repetitions might be required to promote optimal results during the performance of resistance training without external loads.

The contradictory findings for peak and mean EMG activity in the present study might be a consequence of the difficulty to perform maximum contractions during some parts of the movement in the absence of external load, such as when the knees are flexed (this is illustrated on Figure 2). On the other hand, TRT was performed in machines specially designed to promote overload during the full range of motion through its leverage system. It would be important to test if this limitation would lead to disadvantages over the long term and if it would be necessary to restrict the range of motion to the points where muscle activation is higher when training with no external load.

Another interesting point is that the use of visual feedback resulted in no difference for muscle activation when training with no external load, which is in agreement with previous findings [26]. It is possible that the strong verbal incentive provided during the tests was sufficient to induce maximum effort. The similar activation observed with or without visual feedback is an important practical aspect for the use of no external load training in a wide range of situations, since EMG monitoring might not be easily available.

Caution is needed when extrapolating an acute effect to a chronic change. Although the association of muscle activation to muscle strength has been consistently shown [30,31,32,33,34], some authors suggest that muscle activation is not necessarily associated with hypertrophy [34]. However, it has been suggested that mechanotransduction, the translation of mechanical tension into a chemical signal that initiates the cascade responsible for muscle hypertrophy, is likely to occur only in the activated muscle fibers during exercise [35]. This suggests that high levels of muscle activation derived from repeated contractions may be associated with the stimuli that results in increased muscle size [11,36,37,38]. In agreement with this suggestion, previous studies showed a close relation between muscle activation and hypertrophy for both upper [39,40] and lower body muscles [40].

The practical applications of no external load training are promising, especially if we consider the adverse effects of inactivity [41,42,43]. Previous studies have shown that inactivity might be associated with increased adiposity [43] and decreases in muscle strength and loss of muscle mass [44]. Although people are often advised to remain active, many factors might cause apparently unavoidable restriction to physical activity, such as hospitalization, lack of adequate space, unavailability of equipment and public measures such as social isolation and quarantine. In this regard, no load resistance training might be a possible alternative, since it can be performed with minimal space and requires no devices [45]. Even if it cannot be comparable to TRT, as has been shown for some markers of muscle activation in the present study and for gains in muscle strength in healthy young people by Counts et al. [20], resistance training with no external load has been shown to promote high levels of motor unit recruitment and significant increases in muscle mass and strength. Therefore, it can be used to at least mitigate some of the negative effects of physical inactivity. It is important to note the results might depend on individual characteristics, since Counts et al. [20] found limited results for muscle strength in healthy young people, while Barbalho et al. [21] reported similar increases in functionality for hospitalized patients between NL-NF and resistance training with elastic bands. Another possible application of RT with no loads might be in the management of post competition recovery. Considering that athletes usually present decreases in muscle force after games and competitions [46,47,48,49], future studies could investigate the effects of the application of this strategy for increasing the post-competition recovery of muscle function.

It is important to note that resistance with no external load requires high levels of voluntary effort and the participants of the present study were constantly motivated with strong verbal encouragement. It would be interesting to test if no load training would produce significant long-term results when performed without direct supervision and verbal encouragement. One possible caveat of the study might be the involvement of young healthy trained participants, especially when considering its application in clinical settings. However, we chose to recruit healthy people with previous experience in resistance training in order to decrease the risks of this pilot study and avoid the effects of learning during strength testing [47,48]. Moreover, a previous study showed that training status does not influence muscle activation during no load resistance training [26] and, since the participants were not familiar with the no load condition, the results might have been underestimated for this condition.

As a limitation of the study, we can highlight that only the EMG activity of knee extensors in a single exercise was analyzed and caution is required before extrapolating to other muscles and exercises, as well as to long-term adaptations.

## Figures and Tables

**Table 1:** Characteristics of subjects.

Variables	Mean ± Standard Deviation
Age (years)	25.2 ± 3.6
Body mass (kg)	78.9 ± 11.2
Height (m)	1.75 ± 0.06
Body mass index (kg/m^2^)	25.6 ± 3.8
Leg extension experience (months)	34.4 ± 32.4
